# Community-based organizations in the health sector: A scoping review

**DOI:** 10.1186/1478-4505-10-36

**Published:** 2012-11-21

**Authors:** Michael G Wilson, John N Lavis, Adrian Guta

**Affiliations:** 1 McMaster Health Forum, McMaster University, 1280 Main St. West, MML 417, Hamilton, ON, L8S 4L6, Canada; 2 Centre for Health Economics and Policy Analysis, McMaster University, 1280 Main St. West, CRL 209, Hamilton, ON, L8S 4K1, Canada; 3 Department of Clinical Epidemiology and Biostatistics, McMaster University, 1280 Main St. West, Hamilton, ON, L8S 4K1, Canada; 4 Ontario HIV Treatment Network, 1300 Yonge St, Suite 600, Toronto, ON, M4T 1X3, Canada; 5 Department of Political Science, McMaster University, 1280 Main St. West, Hamilton, ON, Canada; 6 Dalla Lana School of Public Health, University of Toronto, Toronto, ON, Canada

## Abstract

Community-based organizations are important health system stakeholders as they provide numerous, often highly valued programs and services to the members of their community. However, community-based organizations are described using diverse terminology and concepts from across a range of disciplines. To better understand the literature related to community-based organizations in the health sector (i.e., those working in health systems or more broadly to address population or public health issues), we conducted a scoping review by using an iterative process to identify existing literature, conceptually map it, and identify gaps and areas for future inquiry.

We searched 18 databases and conducted citation searches using 15 articles to identify relevant literature. All search results were reviewed in duplicate and were included if they addressed the key characteristics of community-based organizations or networks of community-based organizations. We then coded all included articles based on the country focus, type of literature, source of literature, academic discipline, disease sector, terminology used to describe organizations and topics discussed.

We identified 186 articles addressing topics related to the key characteristics of community-based organizations and/or networks of community-based organizations. The literature is largely focused on high-income countries and on mental health and addictions, HIV/AIDS or general/unspecified populations. A large number of different terms have been used in the literature to describe community-based organizations and the literature addresses a range of topics about them (mandate, structure, revenue sources and type and skills or skill mix of staff), the involvement of community members in organizations, how organizations contribute to community organizing and development and how they function in networks with each other and with government (e.g., in policy networks).

Given the range of terms used to describe community-based organizations, this scoping review can be used to further map their meanings/definitions to develop a more comprehensive typology and understanding of community-based organizations. This information can be used in further investigations about the ways in which community-based organizations can be engaged in health system decision-making and the mechanisms available for facilitating or supporting their engagement.

## Background

Community-based organizations are important health system stakeholders as they provide numerous, often highly valued programs and services to the members of their (typically urban) community. In addition, networking and/or developing partnerships between organizations is often particularly important in urban contexts where organizations may need to build coalitions, exchange/share resources, partner, and avoid service duplication [1,2]. Furthermore, community-based organizations often provide services and support to the most marginalized, disadvantaged and stigmatized sections of society [3-11]. For example, community-based organizations in the HIV/AIDS sector often directly provide services, care and resources to many marginalized and/or stigmatized populations including sex workers, drug users, gay men and the homeless [3,6,10]. As Chillag et al. (2002) point out, community-based organizations are well positioned to deliver such services “because they understand their local communities and are connected to the groups they serve” [6]. Similarly, in response to limited access to health services, community-based organizations also often provide essential primary healthcare (especially for the very poor, women and children) in low- and middle-income countries [7,12].

In addition to providing important health services and programs, community-based organizations often play important advocacy roles with the aim of strengthening the health systems in which they work [4,5,12-14]. They are often called upon to collaborate with health system decision-makers and stakeholders in the development of policy, programs and services [15-19], and are increasingly involved in the development and production of research to inform the development of policy, programs and services [20,21]. Such activities help to facilitate the involvement of communities and the public in the planning and implementation of their healthcare, which was a key principle of The Declaration of Alma Ata [22]. Furthermore, successful involvement of community-based organizations (and the public) in decision-making has been shown to increase the likelihood that policies will be appropriate, acceptable and effective [4,23].

While the importance of community-based organizations in health systems has been relatively well articulated, their characteristics are described using diverse terminology and concepts from across a range of disciplines. There appears to be little or no consensus about their nomenclature, core functions, and structure. For instance, in the course of conducting this review, we identified a number of terms that are commonly used to refer to the same, or similar, type of organization, such as those outlined by Bhan et al. (civil society organizations, non-governmental organizations, community-based organizations, faith-based organizations and voluntary organizations) [20]. The descriptors used for community-based organizations may also vary based on the sector or ‘community’ they serve such as specific populations (*e.g.*, AIDS service organizations or community mental health centres). In addition, we also noted during the course of this review that community-based organizations have also been described as a ‘third sector’ or the ‘third way, which refers to the gap filled by these voluntary organizations between what is provided by the state and by the private sector [9,24].

In addition to the varied terms used to describe community-based organizations, there are also several conceptions of what constitute essential organizational features. For instance, in describing the voluntary sector (i.e., the community or non-profit sector), Milligan & Conradson (2006) state that organizations working within this sector “…can be viewed as comprising organizations that are formal, non-profit distributing, constitutionally independent of the state and self-governing”. They further indicate that “While such organizations may employ paid staff and receive funding from the state their remit is to act for public rather than shareholder benefit” [24]. Similarly, Chavis & Florin (1990) assert that voluntary community organizations are geographically based, represent residents of a particular area, volunteer driven, locally initiated and are multi-purpose and flexible allowing them to address a broad array of issues [25] but organizations could also serve communities that are defined beyond geographical terms to include virtual communities or social groups. Others have identified five key characteristics of community-based organizations, indicating they must be: 1) organized (i.e., institutionalized to some degree); 2) separate from government (i.e., private organizations in the sense that they are not run or overseen by a government agency and therefore not part of the public sector); 3) non-profit distributing; 4) self-governing; and 5) voluntary (i.e., some meaningful degree of voluntary participation in the organization’s affairs) [9,26,27]. Therefore, while the terminology may differ (e.g., community-based sector, voluntary sector and third sector), organizations discussed in these sectors have many shared characteristics and perform the same or similar types of activities.

Given the diverse terminology and concepts related to community-based organizations, there is a clear need to assess the extent of the literature related to their key characteristics before undertaking more in-depth analyses of the sector. However, to our knowledge no systematic efforts to identify and outline the existing literature about the characteristics of community-based organizations have been undertaken.

## Objectives

Building on this gap in the literature, our objectives for this scoping review were to:

1. identify existing literature related to the characteristics of community-based organizations and networks of community-based organizations in the health sector (i.e., those working in health systems or more broadly to address population or public health issues);

2. conceptually map the literature according to country focus, sector, discipline, type of literature and topics addressed;

3. identify gaps in the literature and areas for future inquiry that would contribute to a better understanding of the role of community-based organizations in the health sector

## Methods

We used a scoping review to identify, conceptually map and identify gaps in the literature related to the characteristics of community-based organizations and networks of community-based organizations in the health sector. In general, the aims of scoping reviews are to “map *rapidly* the key concepts underpinning a research area and the main sources and types of evidence available, and [they] can be undertaken as stand-alone projects in their own right, especially where an area is complex or has not been reviewed comprehensively before” ([28], emphasis in original). Scoping reviews are often conducted to examine previous research activity, disseminate findings, identify gaps in the research and/or determine the value of conducting a full systematic review [29]. Given the lack of existing comprehensive reviews of this topic and that the literature is likely spread across multiple disciplines and sectors, scoping review methods were ideal for taking the first step towards developing a better understanding of the nature and scope of the literature.

We conducted the scoping review using an iterative process that allowed for flexibility in the search, reviewing and conceptual mapping phases. A flexible approach was important to follow as this area of inquiry is not well-defined and, as a result, important literature may have been omitted if a rigid a priori design was followed. As a result, we developed search terms and inclusion/coding criteria at the initial stages of the review but revised them as the study progressed.

### Literature searches – Phase 1

We first searched 16 databases (see Additional file 1: Appendix 1 for a listing of the databases searched) in Scholars Portal in March 2009 using combination of search terms ([Communit* OR “civil society”] AND [Organiz* OR service OR develop*] AND Health), which yielded 4560 hits. We collectively identified the search terms as relevant descriptors based on our familiarity with the literature at the time. In addition, we conducted a citation search through the Citation Index provided by ISI Web of Knowledge using 22 key articles (see Additional file 1: Appendix 2 for a listing of the articles) that we identified both from our own records and from experts and colleagues. Next, two of us (MGW and AG) initially reviewed a random sample of 200 references from the search results to refine our inclusion criteria and the coding framework (see below for more detailed descriptions of inclusion and coding criteria). Based on our review of the 200 references, we realized that the search was too broad and our selection criteria needed to be narrowed from keeping anything about how communities organize around health-related issues to only articles about the characteristics of community-based organizations or networks of organizations in the health sector (i.e., those working in health systems or more broadly to address population or public health issues).

### Literature searches – Phase 2

During the initial phase of searching and reviewing, we observed that most relevant articles used truncated terms of organi* or mobili* and we therefore included these terms in the following set of revised search terms used in our search of the 16 databases in Scholar’s Portal: (communit* OR “civil society” AND (organi* OR mobili*) AND health (the same databases were searched as in phase 1 – see Additional file 1: Appendix 3). For the citation searches, we eliminated 14 of the 22 references from the original list, which we deemed to be outside the scope of the review (based on our revised inclusion criteria that focused only the characteristics of community-based organizations) and then supplemented the list with 7 citations that we located from the initial review of 200 references (see Additional file 1: Appendix 4 for the results of the revised citation search). These revised searches were conducted in April 2009 with no limits for publication date or language (but the search terms were in English).

We also developed a search strategy for Medline and Embase (bringing our total number of databases searched to 18) after reviewing the results from the Scholars Portal and citation search. The terms from our search of Scholars Portal provided an unmanageable set of results in Medline and Embase (n=46,457) and we therefore developed a revised strategy based on our increased familiarity with the literature. Specifically, we searched Medline and Embase in April 2010 using the following search strategy: community-based organi* OR community organi* OR civil society (again no limits were placed on publication date or language but the search terms were in English).

### Study selection

Two of us (MGW and AG) independently reviewed and applied the selection criteria to all titles and abstracts. Our initial selection criteria were very broad and included any literature related to how communities organize. After reviewing the random sample of 200 titles and abstracts from the original search strategy, we narrowed the criteria to only include literature related to the key characteristics of community-based organizations or networks of community-based organizations which we applied to all search results. During the reviewing process, we marked references as either ‘include’, ‘unclear’ or ‘exclude’ and retrieved the full-text (where possible) for any classified as the former two categories.

### Full-text coding

We iteratively developed a coding framework to conceptually map the included references. One of us (MGW) developed an initial coding framework, which we collectively revised through discussion and after pilot testing it with ten articles. The coding framework included domains related to the country focus, type of literature, source of literature, academic discipline, disease, terminology used to describe organizations and topics discussed (see Additional file 1: Appendix 5 for the full coding framework). For the domain related to the terminology used to describe organizations, we used an outline of five common terms used to describe civil society organizations provided by Bhan et al. (2007) (community-based organizations, non-governmental organizations, civil society organizations, voluntary organizations and faith-based organizations). [20] We then supplemented this list with ‘community mental health centre/organization’ given the large number of articles related to mental health that we noted during title and abstract reviewing.

One of us (MGW) applied the coding framework to the full-text of all included articles, which was then independently checked for consistency and accuracy by another reviewer (AG), with any changes discussed by both reviewers and a final decision arrived at through consensus. The number of articles in each category was then calculated using the reconciled assessments from both reviewers. If no full-text article was available through our respective libraries (McMaster University and the University of Toronto), we coded the article using the title and abstract if they provided sufficient information and documented the articles for which we could not apply the coding framework.

## Results

**Figure 1:**
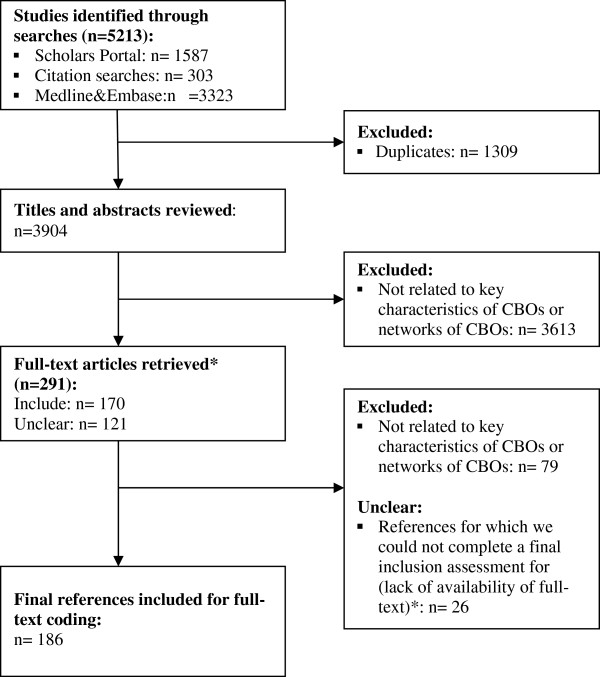
**Flow of study selection.** *We were unable to obtain access to 32 articles due to lack of availability at McMaster or University of Toronto Libraries. For six of these articles, the title and abstract provided sufficient information to determine whether it should be included and to apply the coding framework. As a result, we were unable to complete the coding for 26 articles.

Our searches yielded a total of 5213 references, which was reduced to 3904 after removal of duplicates (see Figure 1 for a summary of the reviewing process). After reviewing the titles and abstracts from the search results, we included 170 and marked 121 as unclear, which we then assessed during full-text coding. Our level of agreement was relatively low with a Kappa of 0.319 (p<0.001), 95% CI (0.264, 0.374). However, all assessments were compared with discrepancies resolved by discussion to ensure consistent application of the inclusion criteria.

Upon reviewing the full-text articles (n=291) we excluded 79 and included *and* coded 186. The remaining 26 articles were not coded as we were unable to retrieve the full-text and the abstracts did not provide enough information to conduct reliable assessments for inclusion and coding. Lists of included articles, excluded articles (after full-text review) and articles that we were unable to code are provided in Additional file 1: Appendices 6,7 and 8 respectively.

We found that just under half of the 186 articles were related to mental health and addictions (n=50, 26.9%) *and/or* HIV/AIDS (n=37, 19.9%) and we present the coding results in Table 1 separately for each of these disease sectors in addition to the overall results. Almost all of the remaining articles addressed a general or unspecified disease sector (e.g. articles discussing organizational structure or the development of networks but not in the context of any specific disease) (n=75, 40.3%) with only three (1.6%) addressing cancer, five (2.7%) cardiovascular disease and two (1.1%) diabetes.

**Table 1:** Results of conceptual mapping of included references

**Coding categories**	**All (n=186)**	**Articles addressing mental health and addictions (n=50)**	**Articles addressing HIV/AIDS (n=37)**
**Country focus**			
i. High-income countries	162 (87.1%)	49 (98.0%)	30 (81.1%)
ii. Low- and middle-income countries	29 (15.6%)	0 (0.0%)	9 (24.3%)
iii. Not clearly stated	3 (1.6%)	1 (2.0%)	0 (0.0%)
**Source of literature**			
i. Journal	172 (92.5%)	39 (78.0%)	36 (97.3%)
ii. Book (whole or chapter)	4 (2.2%)	2 (4.0%)	1 (2.7%)
iii. Report/grey literature	0 (0.0%)	0 (0.0%)	0 (0.0%)
iv. Dissertation	11 (5.9%)	9 (18.0%)	0 (0.0%)
**Type of literature**			
i. Systematic review	1 (0.5%)	0 (0.0%)	0 (0.0%)
ii. Review (not systematic)	8 (4.3%)	3 (6.0%)	3 (8.1%)
iii. Quantitative survey	48 (25.8%)	20 (40.0%)	5 (13.5%)
iv. Qualitative study	48 (25.8%)	15 (30.0%)	8 (21.6%)
iv. Case study	51 (27.4%)	9 (18.0%)	13 (35.1%)
vi. Theory/discussion paper	51 (27.4%)	13 (26.0%)	9 (24.3%)
vii. Commentary/ editorial	12 (6.5%)	2 (4.0%)	2 (5.4%)
viii. Document analysis	4 (2.2%)	2 (4.0%)	2 (5.4%)
**Academic discipline**			
i. Health systems, services and policy	59 (31.7%)	14 (28.0%)	13 (35.1%)
ii. Population and public health	58 (31.2%)	8 (16.0%)	19 (51.4%)
iii. Clinical and epidemiology	20 (10.8%)	6 (12.0%)	5 (13.5%)
iv. Social work	9 (4.8%)	1 (2.0%)	2 (5.4%)
v. Sociology	13 (7.0%)	2 (4.0%)	4 (10.8%)
vi. Political science	11 (5.9%)	2 (4.0%)	3 (8.1%)
vii. Anthropology	3 (1.6%)	2 (4.0%)	0 (0.0%)
viii. Psychology	8 (4.3%)	5 (10.0%)	0 (0.0%)
ix. Organizational/administration	37 (19.9%)	21 (42.0%)	2 (5.4%)
**Terminology used to describe organizations**			
i. Community-based organization	65 (34.9%)	5 (10.0%)	28 (75.7%)
ii. Non-governmental organization	18 (9.7%)	0 (0.0%)	6 (16.2%)
iii. Civil society organization	16 (8.6%)	0 (0.0%)	4 (10.8%)
iv. Voluntary organization	18 (9.7%)	0 (0.0%)	1 (2.7%)
v. Faith-based organization	3 (1.6%)	0 (0.0%)	0 (0.0%)
vi. Community mental health centre/organization	30 (16.1%)	30 (60.0%)	0 (2.6%)
vii. Other†	72 (38.7%)	16 (32.0%)	6 (16.2%)
**Topics discussed**			
i. Organization structure	67 (36.0%)	26 (52.0%)	11 (29.7%)
ii. Organization mandate	95 (51.1%)	21 (42.0%)	21 (73.0%)
iii. Organization type	15 (8.1%)	6 (12.0%)	4 (10.8%)
iv. Community development	10 (5.4%)	3 (6.0%)	2 (5.4%)
v. Community organizing	32 (17.2%)	4 (8.0%)	5 (13.5%)
vi. Community involvement	52 (28.0%)	14 (28.0%)	12 (32.4%)
vii. Community infrastructure	6 (3.2%)	2 (4.0%)	1 (2.7%)
viii. Social movements	2 (1.1%)	1 (2.0%)	2 (5.4%)
ix. Revenue	53 (28.5%)	13 (26.0%)	15 (40.5%)
x. Type/skill of staff	56 (30.1%)	19 (38.0%)	14 (37.8%)
xi. Networks/coalitions	87 (46.8%)	16 (32.0%)	17 (45.9%)
xii. Relationship with government (policy networks)	31 (16.7%)	6 (12.0%)	8 (21.6%)

†See Table 2 for an outline of the terms used to describe organizations in each of the included articles.

Almost all articles focused on high-income countries (n=162, 87.1%) and were published in journals (n=172, 92.5%). However, 24.3% of articles related to HIV/AIDS (n=9) discuss organizations in low- and middle-income countries as compared to 15.6% of all the included articles (n=29). The approach/methods of the articles were varied with most common being discussion or theory-based content (n=51, 27.4%) or empirical findings based on case studies (n=51, 27.4%), qualitative methods (n=48, 25.8%) and quantitative surveys (n=48, 25.8%). Similarly, included articles were from a mix of disciplines with most based in health systems, services and policy literature (n=59, 31.7%), population and public health (n=57, 30.6%), organization and administration (n=37, 19.9%), clinical or epidemiology (n=20, 10.8%).

The included articles presented a wide spectrum of terms to describe community-based organizations. The most common term used from the outline provided by Bhan et al. (2007) was community-based organization (n=66, 34.9%), which was the term used in 75.7% (n=28) of the HIV/AIDS literature. The terms voluntary organization (n=18, 9.7%), non-governmental organization (n=18, 9.7%), civil society organization (n=16, 8.6%) and faith-based organization (n=3, 1.6%) were used less frequently. Community mental health centre/organization was used by 16.1% (n=30) of the articles, which is driven mostly by the fact that a large proportion of the literature was based in mental health and addictions.

More than a third (n=72, 38.7%) of the articles used a term other than one in our coding framework. We documented each additional term (or terms where more than one was presented) used in the included articles and grouped them thematically (see Table 2 for the groupings of terms and Additional file 1: Appendix 9 for a list of all the terms contributing to each grouping). Specifically, we identified eight terms (or very slight variations of terms) that were used in more than one article and an additional eight terms that were used only once. The most popular terms we extracted were related to community coalitions, networks or partnerships (n=20, 10.8%), community health agencies, organizations or centres (n=17, 9.1%), neighborhood associations, congregations, health centers or organizations (n=9, 4.8%), non-profit organizations, agencies, consortium or sector (n=10, 5.4%) or community agencies (n=5, 2.7%).

**Table 2:** Terms used to describe organizations*

**Term**	**Number of articles using term**
Community coalition(s)/networks/partnerships	20
Community health agencies/organizations/centres	17
Non-profit organization/agencies/consortium/sector	10
Neighborhood associations/congregations/health centers/organizations	9
Community agencies	5
Health/social service organization	4
Community development corporation/organization	2
Cooperatives	2
Advocacy organization	1
AIDS service organizations	1
Community care access centers	1
Consumer/survivor initiatives	1
Community boards	1
Third sector organizations	1
Mental health organization	1
Rape crisis center	1

*See Additional file 1: Appendix 5 for a detailed outline of each of the terms included in the groupings of terms outlined.

The topics discussed in the included articles were varied with six of the 13 different topic areas being discussed by at least a quarter of the included articles. Approximately half of the included articles addressed topics related to the mandate or activities of community-based organizations (n=95, 51.1%) or networks/coalitions of organizations (n=87, 46.8%). The other most popular topics addressed were the structure of organizations (n=67, 36.0%), the type or skills of organizational staff (n=56, 30.1%), involvement of community members in the organization (n=52, 28.0%) and sources of revenue (n=53, 28.5%). The topics addressed appear consistent across the HIV/AIDS and mental health and addictions articles except that the former appears to have comparatively more emphasis on revenue sources and the latter tend to focus more on organizational structure and less on networks and coalitions.

## Discussion

### Principal findings

We identified a relatively large number of articles (n=186) addressing topics related to the key characteristics of community-based organizations and/or networks of community-based organizations. The literature that we identified is largely focused on high-income countries and on mental health and addictions, HIV/AIDS or general/unspecified populations of interest. The articles are spread across multiple disciplines with most in health systems, services and policy, population and public health and psychology. A large number of different terms have been used in the literature to describe community-based organizations, which makes it difficult to develop a well-defined outline of organizations and their roles in health systems. Lastly, we found literature related to a range of topics about community-based organizations (mandate, structure, revenue sources and type and skill of staff), the involvement of community members in organizations, how organizations contribute to community organizing and development and how they function in networks with each other and with government (e.g., in policy networks).

### Study meaning

To our knowledge, this is the first attempt to conduct a systematic scoping of the literature related to community-based organizations in the health sector. The results of the review can be used to develop our understandings of the key characteristics of community-based organizations, how they function (individually and in networks), and what roles they are and/or could be playing in health systems. Given the numerous calls over several decades to better engage them in decision-making about health systems [22,30-32], developing a shared understanding of their characteristics and functions is an important and long overlooked step.

This review also complements the existing literature in several ways. First, many of the articles identified in our review discuss topics related to networks, coalitions and/or organizational relationships with government (e.g., policy networks), which provide an important overlap with political theory. The mandate of many community-based organizations often includes advocacy, which frequently takes shape through networks or coalitions of organizations. Depending on how organized a network is, the resources available and the relationship with government (or with other advocacy groups), networks and coalitions of community-based organizations can be important policy actors and influence policy decision-making in a number of ways (e.g., bringing issues onto a government’s agenda and helping to determine whether and how a decision is made). However, the role of advocacy and coalitions needs to problematized because organizations may not always fully represent their communities’ views and, as a result, may advance particular interests, detracting from the democratic and grassroots nature of the organization. Second, involving patients, their families or representatives of patients and their families in the planning or development of healthcare is often highlighted as important activity within health systems [33-36] and community-based organizations can provide opportunities for public engagement in the planning and delivery of programs and services (e.g., through formal governance mechanisms or informal consultative mechanisms and through volunteerism).

The review also revealed two particularly important findings to consider. First, much of the literature is focused on HIV/AIDS and/or mental health and addictions. This is important to note as most people living with or at risk of HIV/AIDS are from marginalized, stigmatized and/or hard-to-reach (and often urban) communities [37-39], from low- and middle-income countries with high rates of HIV incidence and prevalence [40], require complex care and social supports [41] and are underserved with respect to prevention and treatment (especially in low-middle income countries) [40]. Similarly, people with mental health and addictions issues are often stigmatized within society [38], require complex care and social supports [42] and are often hard-to-reach and underserved [39]. As a result, the finding that much of the literature about community-based organizations is focused on HIV/AIDS and mental health and addictions is not entirely surprising as many of these organizations (especially in the HIV/AIDS sector) developed as a grass roots response to gaps in programs and services that governments were not filling and as an advocacy mechanism for broader system level supports. The second notable finding is the lack of literature addressing organizations in the cancer, cardiovascular disease and diabetes sectors as each typically have large networks of charitable and community-based organizations. The minimal amount of literature could be due to a lack of scholarly activity examining the characteristics of community-based organizations or because our search strategy did not include the appropriate terms to identify this literature (see the limitations section below). The third interesting finding to note is the relative lack of literature that is focused on low- and middle-income countries. Given the importance of community-based organizations in the delivery of programs and services and for advocacy in health systems in low- and middle-income countries, the lack of literature is an important gap to fill.

### Strengths and limitations

In addition to the novel contribution of this review, the primary strength is the breadth of the search (18 databases and citation searches using 15 articles) and the rigorous and transparent methods we used to review and code the search results. There main limitation of our review is the potentially limited scope of our search strategy, which stems from the terminology used to describe community-based organizations being broad and difficult to define. Our search strategy focused on the community-based organizations and civil society and, as a result, we may not have captured all relevant literature on this topic. The influence of the scope of our search strategy is reflected in the terminology used to describe organizations that we documented. While the literature we identified used several different terms, most used the term community-based organization, which is likely an indicator of our review not including literature using different terminology to describe the same or similar types of organization. Another potential indicator or our search scope not capturing all facets of the literature is the lack of literature we found related cancer, cardiovascular disease and diabetes despite each having large networks of charitable and community-based organizations. Similarly, we found a lack of literature related to community-based organizations involved in social movements, which further indicates that our search strategy may not have identified all relevant areas of the literature.

### Future research

Given the range of terms used to describe community-based organizations, one area for future research could be to use the findings of this scoping review to further map the meanings/definitions of each in order to develop a more comprehensive typology and understanding of organizations that we describe as community-based or civil society organizations This could involve conducting searches using a revised set of terminology to identify literature that may have been missed in this review. Doing so will help to further develop our understanding of these types of organizations and inform the unique role(s) that community-based organizations are and/or could be playing in the health sector. Building on this, another area for future research is to identify the ways in which community-based organizations should be included in health system decision-making and the mechanisms available for facilitating their engagement. Lastly, future research could focus on examining the impact of different organizational characteristics on the type of activities community-based organizations become involved in. For instance, what impact does the type of funding (e.g., public versus private/commercial), skills of organizational staff, the skill-mix and/or the number of staff and volunteers in the organization have on the types of services and programs provided and the types of advocacy activities (if any) organizations engage in?

## Competing interests

The authors declare that they have no competing interests.

## Authors' contributions

MGW contributed to the conception, design, reviewing, data analysis, wrote the original draft manuscript, and incorporated revisions from each of the co-authors. JNL contributed to the conception and design of the manuscript and provided revisions. AG contributed to the refinement of the original protocol, reviewing, analysis and provided revisions. All authors read and approved the final manuscript.

## Supplementary Material

Additional file 1 Appendices. Click here for file
