# Supplementary material for: Community-based organizations in the health sector: A scoping review
Source: Health Res Policy Syst. 2012 Nov 21;10:36. doi: 10.1186/1478-4505-10-36 (PMC3511187; doi:10.1186/1478-4505-10-36)
Supplement: Additional file 1 — Appendices. [file 1478-4505-10-36-S1.docx]

**Appendix 1**

We developed our search for the scoping review iteratively. We first searched 16 databases in Scholars Portal in March 2009 using combination of search terms ([Communit* OR “civil society”] AND [Organiz* OR service OR develop*] AND Health), which yielded 4560 hits. In table 1, we provide the search terms used, databases searched and a description of the results of the search.

**Table 1: Original Scholars Portal Search**

| **Search Terms** | **Databases** | **Results and description** |
| --- | --- | --- |
| Communit* OR “civil society” (searched using the DE field)  AND  Organiz* OR service OR develop* (searched using the DE field)  AND  Health (searched using the DE field) | 1. ASSIA: Applied Social Sciences Index and Abstracts 2. Health Sciences: A SAGE Full-Text Collection 3. IBSS: International Bibliography of the Social Sciences 4. PAIS Archive 5. PAIS International 6. Political Science: A SAGE Full-Text Collection 7. PsycINFO 8. Science Citation Index Expanded ™ (1976-current) 9. Social Sciences Abstracts 10. Social Sciences Citation Index 11. Social Services Abstracts 12. Sociological Abstracts 13. Sociology: A SAGE Full-Text Collection 14. Urban Studies & Planning: A SAGE Full-Text Collection 15. Urban Studies Abstracts 16. Worldwide Political Science Abstracts | - - This search resulted in 4540 hits, which included peer-reviewed journals, non-peer reviewed journals, conferences and books.   - Deemed the search to be too broad and it needed to be focused more on just community organizations, their mobilization and their key characteristics.   - Based on a review of 200 randomly selected references from the original cited reference search, most relevant articles used terms that contained the truncated terms of organiz* or mobiliz* |

**Appendix 2**

During the first phase of our literature search, we conducted a citation search through the Citation Index provided by ISI Web of Knowledge using 22 key articles that we identified both from our own records and from experts and colleagues. The articles used in the search and the number of hits retrieved from each are outlined in Table 1 below.

**Table 1: Original citation search (rank ordered by number of hits)**

|  | **Reference** | **Number of hits** |
| --- | --- | --- |
| 1 | Etzioni, A. (1993) *The Spirit of Community: the Reinvention of American Society*. New York: Simon & Schuster. Pp.247-267 | 467 |
| 2 | Duncan, J. and Ley, D. (1993) “Introduction: representing the place of culture” in Duncan, J. and Ley, D. (eds) *Place/ Culture/ Representation*. Routledge: London. pp.1-21. | 224 |
| 3 | Eng, E. and E. Parker. 1994. Measuring community competence in the Mississippi Delta: The interface between program evaluation and empowerment. *Health Education Quarterly* 21, no. 2:199-220. | 102 |
| 4 | Gittell, R. and A. Vidal (1998). *Community Organizing: Building Social Capital as a Development Strategy*. Thousand Oaks, CA, Sage. | 101 |
| 5 | Minkler, M. and N. Wallerstein. 1997. Improving health through community organization and community building. In *Community Organizing and Community Building for Health*, ed. Minkler, M., 26-50. (New Brunswick: Rutgers University Press). | 94 |
| 6 | Jewkes, R., & Murcott, A. (1998). Community representativeness: representing the "community"? *Social Science and Medicine*, 46(7), 843-858. | 44 |
| 7 | Hasson, S. & D. Ley (1994) Neighbourhood Organizations and the Welfare State. University of Toronto Press. (Chapter 1: "Neighbourhood organizations, the city, and the state") | 39 |
| 8 | Piven, F. F. and R. A. Cloward (1979). *Poor People's Movements: Why They Succeed, How They Fail*. New York, NY, Vintage Books (Random House). | 35 |
| 9 | Jewkes, R. and A. Murcott (1996) *Meanings of Community*. Social Science and Medicine, 43(4), pp.555-563. | 34 |
| 10 | Popple, K. (1995) *Analysing Community Work: Its Theory and Practice*. Philadelphia, PA: Open University Press. (Chapter 3 & 4) | 17 |
| 11 | Bullock, A. (1990) Community Care: Ideology and Lived Experience, in Ng, R., G, Walker and J. Miller, *Community Organizing and the Canadian State*. Toronto: Garamond Press. | 16 |
| 12 | Campfens, Hubert, ed. (1997). *Community development around the world: Practice, theory, research, training.* Toronto: University of Toronto Press, p.13-46; p.439-469. | 15 |
| 13 | Boutilier, M., S. Cleverly, R Labonte. (2000). “Community as a setting for health promotion”. In B. Poland, L. W. Green and I. Rootman (eds), *Settings For Health Promotion: Linking Theory and Practice*. Thousand Oaks, CA, Sage. | 12 |
| 14 | Lyon, L. (1989) *The Community In Urban Society.* Toronto: Lexington Books. (Chapter 8: "Community development") | 10 |
| 15 | Dixon, J. (1989). "The limits and potential of community development for personal and social change". Community Health Studies, 13(1), 82-92. | 10 |
| 16 | Rabrenovic, Gordana (1996) “Introduction: Economic restructuring, urban change and neighborhoods in crisis” in *Community Builders: A tale of neighborhood mobilization in two cities.* Temple University Press: Philadelphia. | 9 |
| 17 | Mondros, J. and W. Wilson (1994). *Organizing for Power and Empowerment*. New York, NY, Columbia University Press. | 6 |
| 18 | Lotz, Jim (1998). “The origins of community development.” Pp. 113-126 in *The Lichen Factor: The Quest for Community Development in Canada* University College of Cape Breton Press, Sydney NS. | 5 |
| 19 | Rubin, H. J. and I. S. Rubin (1992). *Community Organizing and Development (2nd ed).* New York, NY, Macmillan. | 4 |
| 20 | Lotz, J. (1987). Community development: a short history. Journal of Community Development, May/June, 41-46. | 3 |
| 21 | Stall, S. & Stoecker, R. (1997). Community Organizing or Organizing Community? Gender and the Crafts of Empowerment. Toledo, OH: COMM-ORG Working Paper. | 1 |
| 22 | Murphy, P & J Cunningham. (2003). *Organizing for community controlled development: Renewing civil society*. Thousand Oaks, Calif.: Sage Publications. 339 pp. | 0 |

**Appendix 3**

We revised the database search in second phase of the literature search. The details of the search are outlined in Table 1 below.

**Table 1: Revised Scholars Portal Search**

| **Search Terms** | **Databases and hits** | **Results and description** |
| --- | --- | --- |
| communit* OR “civil society” (searched using the DE field)  AND  organi* OR mobili* (searched using the DE field)  AND  health (searched using the DE field) | 1. ASSIA: Applied Social Sciences Index and Abstracts 2. Health Sciences: A SAGE Full-Text Collection 3. IBSS: International Bibliography of the Social Sciences 4. PAIS Archive 5. PAIS International 6. Political Science: A SAGE Full-Text Collection 7. PsycINFO 8. Science Citation Index Expanded ™ (1976-current) 9. Social Sciences Abstracts 10. Social Sciences Citation Index 11. Social Services Abstracts 12. Sociological Abstracts 13. Sociology: A SAGE Full-Text Collection 14. Urban Studies & Planning: A SAGE Full-Text Collection 15. Urban Studies Abstracts 16. Worldwide Political Science Abstracts | - - This search resulted in 1587 hits, which included peer-reviewed journals, non-peer reviewed journals, conferences and books.   - The only revision to this search was the elimination of the terms ‘service’ and develop* in the second line of the search strategy, which were replaced with mobliz* and mobilis*.   - Search was run in April 2009 |

**Appendix 4**

We revised the papers included in the citation search in second phase of the literature search. The citations from the original search and the newly added citations are outlined in Table 1 below.

**Table 1: Revised citations searched (rank ordered by number of hits for original citations and supplementary citations)**

|  | **Citations kept from original search** | **Number of hits** |
| --- | --- | --- |
| 1 | Gittell, R. and A. Vidal (1998). *Community Organizing: Building Social Capital as a Development Strategy*. Thousand Oaks, CA, Sage. | 101 |
| 2 | Minkler, M. and N. Wallerstein. 1997. Improving health through community organization and community building. In *Community Organizing and Community Building for Health*, ed. Minkler, M., 26-50. (New Brunswick: Rutgers University Press). | 94 |
| 3 | Hasson, S. & D. Ley (1994) Neighbourhood Organizations and the Welfare State. University of Toronto Press. (Chapter 1: "Neighbourhood organizations, the city, and the state") | 39 |
| 4 | Popple, K. (1995) *Analysing Community Work: Its Theory and Practice*. Philadelphia, PA: Open University Press. (Chapter 3 & 4) | 17 |
| 5 | Bullock, A. (1990) Community Care: Ideology and Lived Experience, in Ng, R., G, Walker and J. Miller, Community Organizing and the Canadian State. Toronto: Garamond Press. | 16 |
| 6 | Rabrenovic, Gordana (1996) “Introduction: Economic restructuring, urban change and neighborhoods in crisis” in *Community Builders: A tale of neighborhood mobilization in two cities.* Temple University Press: Philadelphia. | 9 |
| 7 | Rubin, H. J. and I. S. Rubin (1992). *Community Organizing and Development (2nd ed).* New York, NY, Macmillan. | 4 |
| 8 | Stall, S. & Stoecker, R. (1997). Community Organizing or Organizing Community? Gender and the Crafts of Empowerment. Toledo, OH: COMM-ORG Working Paper. | 1 |
|  | **Supplementary citations** | **Number of hits** |
| 9 | Gittell M, Ortega-Bustamante I, Steffy T. Social capital and social change - Women's community activism. Urban Affairs Review 2000;36(2):123-47. | 11 |
| 10 | Stoecker R, Vakil A. States, cultures, and community organizing: Two tales of two neighborhoods. Journal of Urban Affairs 2000;22(4):439-58. | 6 |
| 11 | Saegert S. Building civic capacity in urban neighborhoods: An empirically grounded anatomy. Journal of Urban Affairs 2006;28(3):275-94. | 3 |
| 12 | Bourdages J, Sauvageau L, Lepage C. Factors in creating sustainable intersectoral community mobilization for prevention of heart and lung disease. Health Promotion International 2003;18(2):135-44. | 2 |
| 13 | Nelson DE, Reynolds JH, Luke DA, Mueller NB, Eischen MH, Jordan J et al. Successfully maintaining program funding during trying times: Lessons from tobacco control programs in five states. Journal of Public Health Management and Practice 2007;13(6):612-20. | 0 |
| 14 | Shediac-Rizkallah MC, Bone LR. Planning for the sustainability of community-based health programs: conceptual frameworks and future directions for research, practice and policy. Health Education Research 1998;13(1):87-108. | 0 |
| 15 | East JF. Empowerment through welfare-rights organizing: A feminist perspective. Affilia-Journal of Women and Social Work 2000;15(2):311-28. | 0 |

**Appendix 5**

The framework we used to categorize each of the included references is outlined in table 1 below.

**Table 1: Coding framework for included references**

| **Eligible?**  Eligible for inclusion if it addresses the ways in which community-based organizations function as a single organization or as a network of organizations  🞎 Yes 🞎 No  If yes, then assess reference based on coding categories i-viii below  **i. Information used to code**  🞎 Full-text article 🞎 Title and abstract 🞎 Title only  **ii. Country focus**  🞎 High-income countries 🞎 Low- and middle-income countries  **iii. Type of literature**  🞎 Systematic review (needs to have explicit search and selection criteria)  🞎 Review (not systematic) 🞎 Qualitative study 🞎 Quantitative survey  🞎 Theory/discussion paper 🞎 Commentary/editorial 🞎 Case study  🞎 Other (specify)  **iv. Source of literature**  🞎 Journal 🞎 Book (whole or chapter)  🞎 Grey literature 🞎 Other (specify)  **v. Academic discipline**  🞎 Health services, systems and policy  🞎 Population and public health 🞎 Clinical and Epi  🞎 Social work 🞎 Sociology 🞎 Political science  🞎 Organization/management/administration 🞎 Anthropology  🞎 Psychology 🞎 Other (specify)  **vi. Disease sector**  🞎 HIV/AIDS 🞎 Mental health and addictions  🞎 Cancer 🞎 Cardiovascular disease 🞎 Diabetes  🞎 General/not specified 🞎 Other (specify)  **vii. Terminology used to describe organizations**  🞎 Non-governmental organization 🞎 Voluntary organization  🞎 Community-based organization 🞎 Faith-based organization  🞎 Community mental health centre/organization 🞎 Civil society organization  🞎 CMHC/CMHO  **viii. Topics discussed**  🞎 Organization type/models 🞎 Mandate of CBOs 🞎 Revenue sources  🞎 Geographical focus 🞎 Type/skill of staff 🞎 Community development  🞎 Community infrastructure 🞎 Social movements 🞎 Community organizing  🞎 Networks/coalitions 🞎 Organizational structure  🞎 Community involvement 🞎 Relationship with government (policy networks)  🞎 Other (specify) |
| --- |

**Appendix 6 – Included references**

(1) Adams MS. Becoming an independent community mental health center: Perils of the process. Journal of the National Medical Association 1978;70(7):519.

(2) Aggleton P, Pedrosa JS. Community, Solidarity and Action -- Grupo Pela VIDDA, Brazil. AIDS Care 1994;6(3):343-8.

(3) Agudelo CA. Community participation in health activities: Some concepts and appraisal criteria. Bulletin of the Pan American Health Organization 1983;17(4):375-86.

(4) Alexander JA, Comfort ME, Weiner BJ. Governance in public-private community health partnerships: A survey of the Community Care NetworkSM demonstration sites. Nonprofit Management and Leadership 1998;8(4):311-32.

(5) Alexander JA, Comfort ME, Weiner BJ, Bogue R. Leadership in collaborative community health partnerships. Nonprofit Management and Leadership 2001;12(2):159-76.

(6) Allen SM, Mor V, Fleishman JA, Piette JD. The Organizational Transformation of Advocacy: Growth and Development of AIDS Community-Based Organizations. AIDS & Public Policy Journal 1995;10(1):48-59.

(7) Altman D. The primacy of politics: organizing around AIDS. AIDS 1991;5(Suppl 2):S231-S238.

(8) Altshuler SC, Forward J. The inverted hierarchy: A case manager approach to mental health services. Administration in Mental Health 1978;6(1):57.

(9) Anheier H, Kendall J. Interpersonal trust and voluntary associations: examining three approaches. [Review] [62 refs]. British Journal of Sociology 2002;53(3):343-62.

(10) Arno PS. The nonprofit sector's response to the AIDS epidemic: Community-based services in San Francisco. Am J Public Health 1986;76(11):1325-30.

(11) Ausbrooks RL. An examination of the Administrative Service Delivery and Community Outreach: Components of three urban Community Mental Health Centers: Three case studies. Dissertation Abstracts International: Section B: The Sciences and Engineering 1984;44(10-A):2980.

(12) Azzarto J. Understanding the dynamics of a community health center: From general systems to family systems theory. The Journal of Applied Social Sciences 1994;18(2):183-8.

(13) Bailey ME. Community-based organizations and CDC as partners in HIV education and prevention. Public Health Rep 1991;106(6):702-8.

(14) Balk D. Change in a community mental health center: A case study with twelve lessons. Group and Organization Studies 1978;3(4):447-55.

(15) Banaszak-Holl J, Allen S, Mor V, Schott T. Organizational characteristics associated with agency position in community care networks. Journal of Health and Social Behavior 1998;39(4):368-85.

(16) Barnes N. Collaboration between the U.S. and Mexican HIV/AIDS sectors: The role of community-based organizations and federal HIV/AIDS funding policies in creating a binational political-organizational field. International Journal of Sociology and Social Policy 2002;22(4-6):21-46.

(17) Barnes N. Paradoxes and asymmetries of transnational networks: A comparative case study of Mexico's community-based AIDS organizations. Soc Sci Med 2008;66(4):933-44.

(18) Barton-Villagrana H, Bedney BJ, Miller RL. Peer relationships among community-based organizations (CBO) providing HIV prevention services. Journal of Primary Prevention 2002;23(2):215-34.

(19) Baum F. Public health and civil society: understanding and valuing the connection. Australian & New Zealand Journal of Public Health 1997;21(7):673-5.

(20) Bernstein SM. Exporting philanthropy: Democracy, voluntarism and community organization in post-communist society. Fund Raising Management 1995;26(2):34-7.

(21) Bhan A, Singh JA, Upshur RE, Singer PA, Daar AS. Grand challenges in global health: Engaging civil society organizations in biomedical research in developing countries. PLoS Medicine 2007 September;4(9):e272.

(22) Biagini G. Implications and senses: "AIDS Effects" and civil society. Revista Argentina de Sociologia 2004;2(2):27-42.

(23) Blankenship RL. Collective behavior in organizational settings. Sociology of Work & Occupations 1976;3(2):151.

(24) Blas E, Gilson L, Kelly MP, Labonte R, Lapitan J, Muntaner C et al. Addressing social determinants of health inequities: What can the state and civil society do? Lancet 2008;372(9650):1684-9.

(25) Bockmeyer JL. Devolution and the transformation of community housing activism. Social Science Journal 2003;40(2):175-88.

(26) Boehm A, Litwin H. The influence of organizational and personal characteristics on community planning activity. Administration in Social Work 1997;21(1):31-48.

(27) Borus JF. The coordination of mental health services at the neighborhood level. American Journal of Psychiatry 1975;132(11):1177.

(28) Bourdages J, Sauvageau L, Lepage C. Factors in creating sustainable intersectoral community mobilization for prevention of heart and lung disease. Health Promot Internation 2003;18(2):135-44.

(29) Braithwaite RL, Murphy F, Lythcott N, Blumenthal DS. Community organization and development for health promotion within an urban black community: A conceptual model. Health Education 1989;20(5):56-60.

(30) Brandon W. Politics, administration, and conflict in neighborhood health centers. Journal of Health Politics, Policy and Law 1977;2(1):79-99.

(31) Briggs HE, Koroloff NM. Enhancing family advocacy networks: An analysis of the roles of sponsoring organizations. Community Mental Health Journal 1995;31(4):317.

(32) Brooks F. Resolving the dilemma between organizing and services: Los Angeles ACORN's welfare advocacy. Social Work 2005;50(3):262-70.

(33) Broskowski A, Peterson DR, Fishman DB. Assessment and decision-making in community mental health centers. Assessment for Decision. Rutgers Symposia on Applied Psychology, Vol. 1.New Brunswick, NJ, US: Rutgers University Press; 1987. p. 321-47.

(34) Brown ER. Community organization influence on local public health care policy: A general research model and comparative case study. Health Education Quarterly 1983;10(3-4):205-33.

(35) Buchanan DR. Building Academic-Community Linkages for Health Promotion: A Case Study in Massachusetts. American Journal of Health Promotion 1996;10(4):262-9.

(36) Burkett TL, Gately R, Martin P. The effectiveness of interorganizational rape processing networks: A comparative study of twenty-two communities. Southern Sociological Society. 2001.

(37) Burney LE. Community organization: An affective tool. American Journal of Public Health & the Nation's Health 1954;44(1):1-6.

(38) Butterfoss FD, Goodman RM, Wandersman A. Community coalitions for prevention and health promotion: Factors predicting satisfaction, participation, and planning. Health Education Quarterly 1996;23(1):65.

(39) Butterfoss FD. Coalitions and partnerships in community health. San Francisco, CA, US: Jossey-Bass; 2007.

(40) Byington DB, Martin DY, DiNitto DM, Maxwell MS. Organizational affiliation and effectiveness: The case of rape crisis centers. Administration in Social Work 1991;15(3):83-103.

(41) Byrne A, Hansberry J. Collaboration: Leveraging resources and expertise. New Directions for Youth Development 2007;114:75-84.

(42) Carey GE, Braunack-Mayer AJ. Exploring the effects of government funding on community-based organizations: 'Top-down' or 'bottom-up' approaches to health promotion? Global Health Promotion 2009;16(3):45-52.

(43) Carline JD, Patterson DG. Characteristics of health professions schools, public school systems, and community-based organizations in successful partnerships to increase the numbers of underrepresented minority students entering health professions education. Academic Medicine 2003;78(5):467-82.

(44) Carruthers S. The organization of a community: Community-based prevention of injecting drug use-related health problems. Substance Use & Misuse 2007;42(12-13):1971-7.

(45) Chavis DM, Florin P. Nurturing grassroots initiatives for health and housing. Bulletin of the New York Academy of Medicine 1990;66(5):558-72.

(46) Chavis DM. The paradoxes and promise of community coalitions. Am J Community Psychol 2001;29(2):321-9.

(47) Chillag K, Bartholow K, Cordeiro J, Swanson S, Patterson J, Stebbins S et al. Factors affecting the delivery of HIV/AIDS prevention programs by community-based organizations. AIDS Education and Prevention 2002;14(3):27.

(48) Clark RE, Dorwart RA, Epstein SS. Managing competition in public and private mental health agencies: Implications for services and policy. Milbank Quarterly 1994;72(4):653.

(49) Clifford DL. A consideration of simple measures and organizational structure. Evaluation & Program Planning 1987;10(3):231.

(50) Crampton P, Davis P, Lay-Yee R. Primary care teams: New Zealand's experience with community-governed non-profit primary care. Health Policy 2005;72(2):233-43.

(51) Creese G. Government restructuring and settlement agencies in Vancouver: Bringing advocacy back in. In: Conradson D, Milligan C, editors. Landscapes of Voluntarism; New Spaces of Health, Welfare and Governance.Bristol, UK: Policy Press; 2006.

(52) Dagirmanjian S. The work experience of service staff in mental health service organizations and its relationship to leadership style and organizational structure. Dissertation Abstracts International: Section B: The Sciences and Engineering 1982;43(5-B):1609.

(53) Dearing JW, Larson RS, Randall LM. Local reinvention of the CDC HIV prevention community planning initiative. Journal of Community Health 1998;23(2):113-26.

(54) DeSouza R, Jyoti Dutta M. Global and local networking for HIV/AIDS prevention: The case of the Saathii e-forum. J Health Commun 2008;13(4):326-44.

(55) Dill A. Institutional environments and organizational responses to AIDS. Journal of Health and Social Behavior 1994;35(4):349-69.

(56) Doyle C, Patel P. Civil society organisations and global health initiatives: Problems of legitimacy. Soc Sci Med 2008;66(9):1928-38.

(57) Dunlop JM, Angell GB. Inside-outside: Boundary-spanning challenges in building rural health coalitions. Professional Development: The International Journal of Continuing Social Work Education 2001;4(1):40-8.

(58) Edwards B, Woods M. Voluntarism and new forms of governance in rural communities. In: Bristol U, editor. Landscapes of Voluntarism; New Spaces of Health, Welfare and Governance.Policy Press: 2006.

(59) Eiel C. Survival starts at the top. Behavioral Health Management 1997;17:32-4.

(60) Eilbert KW, Lafronza V. Working together for community health--A model and case studies. Evaluation & Program Planning 2005;28(2):185.

(61) Eilenberg J, Townsend EJ, Oudens E. Who's in charge here anyway? Managing the management split in mental health organizations. Administration and Policy in Mental Health 2000;27(5):287.

(62) El Ansari W, Phillips CJ. Interprofesional collaboration: A stakeholder approach to evaluation of voluntary participation in community partnerships. Journal of Interprofessional Care 2001;15(4):351-68.

(63) Elliott SJ, Jolin MA, Walker R. Partnering in and for heart health promotion: findings from a survey of community organizations. Canadian Journal of Public Health 2000;91(3):229-33.

(64) Emanoil P. The key to public health is community: the movement called community health means more than just access to health care. Human Ecology 2000;28(2):16-8.

(65) Feinberg ME, Greenberg MT, Osgood DW. Readiness, functioning, and perceived effectiveness in community prevention coalitions: A study of communities that care. Am J Community Psychol 2004;33(3/4):163-76.

(66) Fennell ML, Allen SM, Laliberte L. Community-based service providers for people with chronic care needs: Survival in an uncertain environment. Research in Social Problems and Public Policy 2001;8:43-57.

(67) Ferrinho P, Robb D, Cornielje H, Rex G. Primary health care in support of community development. World Health Forum 1993;14(2):158-62.

(68) Fleishman JA, Mor V, Piette JD, Allen SM. Organizing AIDS service consortia - lead agency identity and consortium cohesion. Soc Serv Rev 1992;66(4):547-70.

(69) Ford JP. Decision-making in community mental health centers: An exploratory study. Dissertation Abstracts International, A: The Humanities and Social Sciences 1987;47(10):3872.

(70) Foster-Fishman PG, Berkowitz SL, Lounsbury DW, Jacobson S, Allen NA. Building collaborative capacity in community coalitions: A review and integrative framework. Am J Community Psychol 2001;29(2):241.

(71) Fournier D, Rene JF, Garon S, Fontaine A, Chenard J, Lefebvre C. The Partnership Dynamic among Community Organizations in the Context of the Reorganization of Health and Social Services. Nouvelles Practiques Sociales 2001;14(1):111-31.

(72) Franklin JL, Kittredge LD. Organizational problems in community mental health centers. Administration in Mental Health 1975;Spring:60.

(73) Freudenberg N, Kohn S. The Washington Heights health action project: A new role for social service workers in community organizing. Catalyst 1982;4(1):7-23.

(74) Freudenberg N. Citizen action for environmental health: Report on a survey of community organizations. Am J Public Health 1984;74:444-8.

(75) Freudenberg N. Community organization, housing, and health: A perspective for public health workers. Bulletin of the New York Academy of Medicine 1990;66(5):451-62.

(76) Freudenberg N, Trinidad U. The role of community organizations in AIDS prevention in two Latino communities in New York City. Health Education Quarterly 1992;19(2):219-32.

(77) Gabbert JP. Community mental health centers and the problems of self-sufficiency: An application of theories of organizational adaptation to the environment. Dissertation Abstracts International: Section B: The Sciences and Engineering 1981;41(12-B, Pt 1):4725.

(78) Gamm LD. Advancing community health through community health partnerships. Journal of Healthcare Management 1998;43(1):51-66.

(79) Gaumer B, Fleury MJ. CLSCs in Quebec: Thirty years of community action. Social Work in Public Health 2008;23(4):89-106.

(80) Gee L, Smucker DR, Chin MH, Curlin FA. Partnering together? Relationships between faith-based community health centers andneighborhood congregations. Southern medical journal 2005;98(12):1245-50.

(81) Gell F. Building relations with women's organisations in relief work. Links 1997;6-7.

(82) Gentry D, Rundall TG. Staffing in AIDS service organizations: The volunteer contribution. Journal of Health & Human Services Administration 1995;18(2):190-204.

(83) Gittell M, Ortega-Bustamante I, Steffy T. Social capital and social change: Women's community activism. Urban Aff Rev 2000;36(2):123-47.

(84) Gittell R, Wilder M. Community development corporations: Critical factors that influence success. Journal of Urban Affairs 1999;21(3):341-61.

(85) Gomez-Jauregui J. The feasibility of government partnerships with NGOs in the reproductive health field in Mexico. Reproductive Health Matters 2004;12(24):42-55.

(86) Goodstein LD. Organizational development as a model for community consultation. Hospital & Community Psychiatry 1972;23(6):165.

(87) Granner ML, Sharpe PA. Evaluating community coalition characteristics and functioning: A summary of measurement tools. Health Education Research 2004;19(5):514-32.

(88) Green LW, Kreuter MW. Are community organization and health promotion one process or two? American Journal of Health Promotion 1993;7(3):221.

(89) Grenier G, Fleury MJ. Mental health community organizations in Quebec: Role and partnership models. Sante Mentale au Quebec 2009;34(1):101-26.

(90) Griffith JR. The strategic agenda for community health care organizations. Health Care Management Review 1997;22(3):82-91.

(91) Grusky O. The organization and effectiveness of community mental health systems. Administration and Policy in Mental Health 1995;22(4):361.

(92) Gulzar L, Henry B. Interorganizational collaboration for health care between nongovernmental organizations (NGOs) in Pakistan. Soc Sci Med 2005;61(9):1930-43.

(93) Habasch R. The Palestinian Authority and Civil Society: A Case Study of Women's and Health Organizations in the West Bank. Dissertation Abstracts International, A: The Humanities and Social Sciences 2001;61(8):3329.

(94) Hall J. Productivity improvement through team building and organizational redevelopment: Evaluating the experiences of a human services agency at the county level. Public Personnel Management 1985;14(4):409.

(95) Hallock AC, Vaughan WTJr. Community organization: A dynamic component of community mental health practice. American Journal of Orthopsychiatry 1956;26(4):691-706.

(96) Halseth G, Williams A. Guthrie house: A rural community organizing for wellness. Health & Place 1999;5(1):27-44.

(97) Harper GW, Bangi AK, Contreras R, Pedraza A, Tolliver M, Vess L. Diverse phases of collaboration: Working together to improve community-based HIV interventions for adolescents. Am J Community Psychol 2004;33(3-4):193-204.

(98) Harvey P. Rehabilitation in complex political emergencies: Is rebuilding civil society the answer? Disasters 1998;22(3):200-17.

(99) Hasnain-Wynia R, Margolin FS, Bazzoli GJ. Models for community health partnerships. Health Forum Journal 2001;44(3):29-33.

(100) Hasson S, Ley D. Neighborhood organizations, the welfare state, and citizenship rights. Urban Aff Rev 1997;33(1):28-58.

(101) Hill A, De Zapien JG, Staten LK, McClelland DJ, Garza R, Moore-Monroy M et al. From program to policy: Expanding the role of community coalitions. Preventing Chronic Disease 2007;4(4):A103.

(102) Hooyman NR. The practice implications of interorganizational theory for services integration. Journal of Sociology & Social Welfare 1976;3(5):558.

(103) Huang K. The relationship between embeddedness and organizational social performance in a community mental health network under managed care. Dissertation Abstracts International, A: The Humanities and Social Sciences 2006;66(8):3089.

(104) Hughey J, Peterson NA, Lowe JB, Oprescu F. Empowerment and sense of community: Clarifying their relationship in community organizations. Health Education & Behavior 2008;35(5):651-63.

(105) Ikegami C. HIV prevention and community-based organizations in Japan. Journal of Acquired Immune Deficiency Syndromes & Human Retrovirology 1997;14(Suppl 2):S51-S57.

(106) Indyk D, Rier DA. Wiring the HIV/AIDS System: Building Interorganizational Infrastructure to Link People, Sites, and Networks. Soc Work Health Care 2006;42(3-4):29-45.

(107) Isett KR, Provan KG. The evolution of dyadic interorganizational relationships in a network of publicly funded nonprofit agencies. Journal of Public Administration and Theory 2005;15(1):149-65.

(108) Iurato JM. Leadership trust and member motivation in community mental health organizations. Dissertation Abstracts International: Section B: The Sciences and Engineering 2007;68(1-B):659.

(109) Jackson ED. Comprehensive community mental health center-business community relationship: A reexamination. Community Mental Health Journal 1977;13(1):68.

(110) Janzen R, Nelson G, Trainor J, Ochocka J. A longitudinal study of mental health consumer/survivor initiatives: Part 4--Benefits beyond the self? A quantitative and qualitative study of system-level activities and impacts. J Community Psychol 2006;34(3):285.

(111) Jareg P, Kaseje DCO. Growth of civil society in developing countries: Implications for health. Lancet 1998;14:819-22.

(112) Jerome GV, Hedges JR, Mann NC. A structured approach to developing a community organization database. Academic Emergency Medicine 1996;3(10):984-7.

(113) Jerrell JM, Larsen JK. Policy Shifts and Organizational Adaptation: A Review of Current Developments. Community Mental Health Journal 1984;20(4):282-93.

(114) Jetté C, Mathieu RA, Dumais L. Analysis of the social impact of the non-profit sector in four districts of Montreal. Nouvelles Practiques Sociales 2002;15(2):87-103.

(115) Johnston PT, Charfauros EA, Galatowitsch P, Khoshnood K, Miller J, Sullivan L. Remaking AIDS response: Politically regulated social reproduction and some problems in AIDS response work. American Sociological Association, 1991 1991.

(116) Katoff L. Community-based services for people with AIDS. Primary Care 1992;19(1):231-43.

(117) Kegler MC. Factors that contribute to effective community health promotion coalitions: A study of 10 Project ASSIST coalitions in North Carolina. Health Education & Behavior 1998;25(3):338.

(118) Kelly CM, Baker EA, Williams D, Nanney MS, Haire-Joshu D. Organizational capacity's effects on the delivery and outcomes of health education programs. Journal of Public Health Management & Practice 2004;10(2):164-70.

(119) Klein SJ, Birkhead GS, Murphy DP. Role of community-based organizations in control of sexually transmitted diseases. JAMA 1998;280(5):419-20.

(120) Kloos H, Haile Mariam D. Community-Based Organizations and Poverty Alleviation Programs in HIV/AIDS Prevention and Control in Ethiopia: A Preliminary Survey. Northeast African Studies 2000;7(2):13-33.

(121) Kramer JS, Philliber S, Brindis CD, Kamin SL, Chadwick AE, Revels ML et al. Coalition models: Lessons learned from the CDC's Community Coalition Partnership Programs for the Prevention of Teen Pregnancy. Journal of Adolescent Health 2005;37(3 Suppl):S20-S30.

(122) Lariviere C. Necessary development of partnerships: Is interdependence a threat to autonomy? Nouvelles Practiques Sociales 2001;14(1):64-80.

(123) Lemieux-Charles L, Chambers LW, Cockerill R, Jaglal S, Brazil K, Cohen C et al. Evaluating the effectiveness of community-based dementia care networks: The dementia care networks' study. The Gerontologist 2005;45(4):456.

(124) Lenk KM, Toomey TL, Wagenaar AC, Bosma LM, Vessey J. Can neighborhood associations be allies in health policy efforts? Political activity among neighborhood associations. J Community Psychol 2002;30(1):57.

(125) Levine S, White PE. Exchange as a conceptual framework for the study of interorganizational relationships. Administrative Science Quarterly 1961;5(4):583-601.

(126) Lewis J. Voluntary organizations in "new partnership" with local authorities: The anatomy of a contract. Social Policy and Administration 1994;28(3):206-20.

(127) Libby MK, Austin MJ. Building a coalition of non-profit agencies to collaborate with a county health and human services agency: The Napa county behavioral health committee of the Napa coalition of non-profits. Administration in Social Work 2002;26(4):81.

(128) Magrath I. Role of the global civil society. Lancet 2005;366(9486):613-5.

(129) Marwell NP. Privatizing the welfare state: Nonprofit community-based organizations as political actors. Am Sociol Rev 2004;69(2):265-91.

(130) Maupin JN. 'Fruit of the accords': Healthcare reform and civil participation in Highland Guatemala. Soc Sci Med 2009;68(8):1456-63.

(131) May ML, Contreras RB. Promotor(a)s, the organizations in which they work, and an emerging paradox: How organizational structure and scope impact promotor(a)s' work. Health Policy 2007;82(2):153.

(132) Mayberry RM, Daniels P, Akintobi TH, Yancey EM, Berry J, Clark N. Community-based organizations' capacity to plan, implement, and evaluate success. Journal of Community Health 2008;33(5):285-92.

(133) Mayberry RM, Daniels P, Yancey EM, Akintobi TH, Berry J, Clark N et al. Enhancing community-based organizations' capacity for HIV/AIDS education and prevention. Evaluation & Program Planning 2009;32(3):213-20.

(134) McAlearney JS, McAlearney AS. Community Health Center Integration: Experience in the State of Ohio. J Health Care Poor Underserved 2006;17(1):55-64.

(135) McFall S, Norton BL, McLeroy KR. A qualitative evaluation of rural community coalitions. International Quarterly of Community Health Education 2004;23(4):311.

(136) McKinney MM, Bragg K. Building an HIV care network in central Iowa. AIDS & Public Policy Journal 1994;9(3):114-22.

(137) McNabola FM. Organizational analysis of community mental health centers. Dissertation Abstracts International: Section B: The Sciences and Engineering 1973;34(4-B):1615.

(138) McRae J, Lawlor L, Nelson B. Counteracting bureaucratic resistance in welfare and mental health -- A working agreement approach. Administration in Mental Health 1984;12(2):123-32.

(139) Mercer K. Facilitating organizational mergers: Amalgamation of community care access centres. Journal of Health Services Research & Policy 2008;13:46-51.

(140) Messal JL. Organizational growth and change: The life cycle of a community mental health center. Administration in Mental Health 1980;8(1):12-22.

(141) Milio N. Health care organizations and innovations. Journal of Health and Social Behavior 1971;12(2):163-73.

(142) Miller LD, Moore LR. Developing statewide consumer networks. Psychiatric Services 2009;60(3):291-3.

(143) Miller RL. Innovation in HIV prevention: Organizational and intervention characteristics affecting program adoption. Am J Community Psychol 2001;29(4):621-47.

(144) Milligan C, Fyfe NR. Putting the voluntary sector in its place: Geographical perspectives on voluntary activity and social welfare in Glasgow. J Soc Policy 2004;33:73-93.

(145) Milward HB, Provan KG. Principles for controlling agents: the political economy of network structure. Journal of Public Administration and Theory 1998;8(2):203-21.

(146) Mitchell SM, Shortell SM. The governance and management of effective community health partnerships: A typology for research, policy, and practice. The Milbank Quarterly 2000;78(2):241-89.

(147) Mitzen P. Organizational ethics in a nonprofit agency: Changing practice, enduring values. Generations 1998;22(3):102-4.

(148) Mogulof MB. Advocates for themselves: Citizen participation in Federally supported community organizations. Community Mental Health Journal 1974;10(1):66-76.

(149) Morris A, Bloom JR. Contextual factors affecting job satisfaction and organizational commitment in community mental health centers undergoing system changes in the financing of care. Mental Health Services Research 2002;4(2):71.

(150) Morris S. Defining the nonprofit sector: Some lessons from history. Voluntas: International Journal of Voluntary and Nonprofit Organizations 2000;11(1):25-43.

(151) Morrissey JP, Tausig M, Lindsey ML. Community mental health delivery systems: A network perspective. American Behavioral Scientist 1985;28(5):704.

(152) Morton LW. The contributions of business and civil society sectors to rural capacity to solve local health issues. Journal of Rural Health 2001;17(3):167-78.

(153) Myers AM, Pfeiffle P, Hinsdale K. Building a community-based consortium for AIDS patient services. Public Health Rep 1994;109(4):555-62.

(154) Nathan S, Rotem A, Ritchie J. Closing the gap: Building the capacity of non-government organizations as advocates for health equity. Health Promot Internation 2002;17(1):69-78.

(155) Nu'Man J, King W, Bhalakia A, Criss S. A framework for building organizational capacity integrating planning, monitoring, and evaluation. Journal of Public Health Management & Practice 2007;(Suppl):S24-S32.

(156) O'Leary JJ. A case study and analysis of a human service organization using Domain Theory of organizational behavior. Dissertation Abstracts International: Section B: The Sciences and Engineering 1985;46(1-B):336.

(157) Ochocka J, Nelson G, Lord J. Organizational change towards the empowerment-community integration paradigm in community mental health. Canadian Journal of Community Mental Health 1999;18(2):59.

(158) Ouellette PM. Action leadership: the development of an approach to leadership enhancement for grassroots community leaders in children's mental health. Journal of Behavioral Health Services & Research 1999;26(2):171-84.

(159) Ozcan YA, Shukla RK, Tyler LH. Organizational performance in the community mental health care system: The need fulfillment perspective. Organization Science 1997;8(2):176.

(160) Palermo T, Ehlers J. Coalitions: Partnerships to promote agricultural health and safety. Journal of Agricultural Safety & Health 2002;8(2):161-74.

(161) Pazaratz D. The establishment and growth of a mental health center. Residential Treatment for Children & Youth 1998;15(4):11-23.

(162) Penner S. A study of coalitions among HIV/AIDS service organizations. Sociological Perspectives 1995;38:217-39.

(163) Podsiadlo M. Social defenses in a large community mental health organization. Dissertation Abstracts International: Section B: The Sciences and Engineering 2004;65(6-B):3208.

(164) Poole D. Achieving national health goals in prevention with community organization: The "bottom up" approach. Journal of Community Practice 1997;4(2):77-92.

(165) Provan KG, Milward HB. A preliminary theory of interorganizational network effectiveness: A comparative study of four community mental health systems. Administrative Science Quarterly 1995;40(1):1.

(166) Provan KG, Kenis P. Modes of network governance: Structure, management, and effectiveness. Journal of Public Administration and Theory 2008;18(2):229-52.

(167) Robins L, Backstrom C. Organizational imperatives and policy perspectives of AIDS community-based organizations: A view from the states. AIDS & Public Policy Journal 1999;14(1):3-19.

(168) Rodriguez R, Frohlich KL. The role of community organizations in the transformation of the health services delivery system in the Montreal metropolitan area. Canadian Journal of Public Health 1999;90(1):41-4.

(169) Rousseau C. Community empowerment: The alternative resources movement in Quebec. Community Mental Health Journal 1993;29(6):535-46.

(170) Roy CM, Cain R. The involvement of people living with HIV/AIDS in community-based organizations: Contributions and constraints. AIDS Care 2001;13(4):421-32.

(171) Rutledge R, Robinson L. Community-based organizations are critical partners in providing complete cancer care. Current Oncology 2009;16(2):29-33.

(172) Schurmann AT, Mahmud S. Civil society, health, and social exclusion in Bangladesh. Journal of Health, Population & Nutrition 2009;27(4):536-44.

(173) Secker J, Hill K. Broadening the partnerships: Experiences of working across community agencies. Journal of Interprofessional Care 2001;15(4):341.

(174) Seckinelgin H. Who can help people with HIV/AIDS in Africa? Governance of HIV/AIDS and civil society. Voluntas 2004;15(3):287-304.

(175) Singer HH, Kegler MC. Assessing interorganizational networks as a dimension of community capacity: Illustrations from a community intervention to prevent lead poisoning. Health Education & Behavior 2004;31(6):808-21.

(176) Steedman E, Rabinowicz J. Changing dynamics in the Canadian voluntary sector: Challenges in sustaining organizational capacity to support healthy communities. The Journal Of The Royal Society For The Promotion Of Health 2006;126(6):275-9.

(177) Stephens KK, Rimal RN, Flora JA. Expanding the reach of health campaigns: Community organizations as meta-channels for the dissemination of health information. J Health Commun 2004;9(Suppl 1):97-111.

(178) Suarez-Balcazar Y, Hellwig M, Kouba J, Redmond L, Martinez L, Block D et al. The making of an interdisciplinary partnership: The case of the Chicago Food System Collaborative. Am J Community Psychol 2006;38(1-2):113-23.

(179) Takahashi LM, Smutny G. Collaboration among small, community-based organizations: Strategies and challenges in turbulent environments. Journal of Planning Education and Research 2001;21(2):141-53.

(180) Takahashi LM, Smutny G. Collaborative windows and organizational governance: Exploring the formation and demise of social service partnerships. Nonprofit Voluntary Sector Q 2002;31(2):165-85.

(181) Vanier C, Fortin D. Mental health resources in the community: Development of a typology. Canadian Journal of Community Mental Health 1997;16(1):87-103.

(182) Vincent J, Harrow J. Comparing thistles and roses: The application of governmental-voluntary sector relations theory to Scotland and England. Voluntas 2005;16(4):375-95.

(183) Wahl E. The role of community organizations. Women's Health Issues 1994;4(2):78-80.

(184) Walker R. Inter-Organizational Linkages as Mediating Structures in Community Health. Health Promot Internation 1992;7(4):257-64.

(185) Wandersman A, Valois R, Ochs L, De la Cruz DS, Adkins E, Goodman RM. Toward a social ecology of community coalitions. American Journal of Health Promotion 1996;10(4):299-307.

(186) Wetta-Hall R, Ablah E, Oler-Manske J, Berry M, Molgaard C. Strategies for community-based organization capacity building: Planning on a shoestring budget. Health Care Manager 2004;23(4):302-9.

(187) White PE, Levin L, Levine S. Community health organizations and resources. Handbook of Medical Sociology. 3rd ed. Englewood Cliffs, N.J.: Prentice-Hall; 1973. p. 347-68.

(188) Wickizer TM, Von Korff M, Cheadle A. Activating communities for health promotion: A process evaluation method. Am J Public Health 1993;83:561-7.

(189) Wilson PA. Interorganizational relationships and coordination of health care services: A study of three neighborhood health centers. Dissertation Abstracts International: Section B: The Sciences and Engineering 1976;36(12-A):8303.

(190) Wu FS. International non-governmental actors in HIV/AIDS prevention in China. Cell Research 2005;15(11-12):919-22.

**Appendix 7 – Excluded articles (from full-text review)**

(1) Anderson JA, McIntyre JS, Rotto KI, Robertson DC. Developing and maintaining collaboration in systems of care for children and youths with emotional and behavioral disabilities and their families. American Journal of Orthopsychiatry 2002;72(4):514-25.

(2) Anonymous. Community organizing and community building for health. Journal of Social Work Education 1998;34(1):137.

(3) Anonymous. Community groups push for a greater role in stemming epidemic: Barriers, including lack of funding, still exist. AIDS Alert 18(11):133, 135, 137, 2003.

(4) Anspach RR. Everyday methods for assessing organizational effectiveness. Soc Probl 1991;38:1-179.

(5) Archibald ME. The population dynamics of modern self-help/mutual-aid: Organizational and institutional change in the civil sector, 1955-2000. Dissertation Abstracts International, A: The Humanities and Social Sciences 2003;63(8):3020.

(6) Aviram U. Community care of the mentally ill: Continuing problems and current issues. Community Mental Health Journal 1990;26(1):69.

(7) Baker F. Are community mental health centers organizing for continuity and efficiency? In: Harshbarger D, Maley RF, editors. Behavior analysis and systems analysis: An integrative approach to mental health programs.Oxford, England: Behaviordelia; 1974.

(8) Baker JR. Neighborhood Organizations and the Welfare-State (Review of Hasson,S, Ley,D). Urban Aff Rev 1995;30(6):880-7.

(9) Baldwin DM. Meeting production: The economics of contracting mental illness. Social Science & Medicine 1990;30(9):961.

(10) Barbour WC. Community based organizations: The good, the bad and the impact, if accreditation becomes an issue. Journal of Vocational Rehabilitation 1997;9(3):217-23.

(11) Barret JP. The art of administration as it relates to hospitals: The community health organizations and other community organizations. Hospital Management 1960;90:32-4.

(12) Barry F. Mobilization of community resources to work with abusive and neglectful families: Community organization in New York State. Child Abuse & Neglect 1982;6(2):177-84.

(13) Bhugra D, La GJ. Community organisations' expectations of mental health statutory services. Irish Journal of Psychological Medicine 1997;14(2):57-9.

(14) Bigelow B, Stone MM. Why don't they do what we want? An exploration of organizational responses to institutional pressures in community health centers. Public Administration Review 1995;55(2):183.

(15) Black BJ. Comprehensive community mental health services: Setting social policy. Social Work 1967;12(1):51.

(16) Brinkerhoff DW, Goldsmith AA. Promoting the sustainability of development institutions: A framework for strategy. World Dev 1992;20(3):369-83.

(17) Brown A. Redesigning patient services. Nursing Management 2006;13(2):26-30.

(18) Burgess JH. Goals versus process orientation policy in community mental health. Social Psychiatry 1975;10(1):9.

(19) Curlin FA, Serrano KD, Baker MG, Carricaburu SL, Smucker DR, Chin MH. Following the call: How providers make sense of their decisions to work in faith-based and secular urban community health centers. J Health Care Poor Underserved 2006;17(4):944-57.

(20) D'Aunno T, Price RH. Organizational adaptation to changing environments: Community mental health and drug abuse services. American Behavioral Scientist 1985;28(5):669-83.

(21) Davidson R, Hunter S. Community care in practice. Social Policy and Administration 1995;29(1):69-73.

(22) Dhooper SS. Social Work in Health Care in the 21st Century. Thousand Oaks, CA, US: Sage Publications; 1997.

(23) Doherty WJ, Mendenhall TJ. Citizen health care: A model for engaging patients, families, and communities as coproducers of health. Families, Systems and Health 2006;24(3):251-63.

(24) Donaldson A, Lank E, Maher J. Connecting through communities: How a voluntary organization is influencing healthcare policy and practice. Journal of Change Management 2005;5(1):71.

(25) Donaldson A, Lank E, Maher J. Making the invisible visible: How a voluntary organization is learning from its work with groups and communities. Journal of Change Management 2005;5(2):191.

(26) Eaddy ML, Cooper S, Lentner TH. The "private" community mental health organization. In: Cooper S, Lentner TH, editors. Innovations in Community Mental Health.Sarasota, FL, US: Professional Resource Press/Professional Resource Exchange; 1992. p. 51-72.

(27) Edmundo K, Guimaraes W, Vasconcelos MS, Baptista AP, Becker D. Network of communities in the fight against AIDS: Local actions to address health inequities and promote health in Rio de Janeiro, Brazil. Promotion & Education 2005;(Suppl 3):15-9.

(28) Elder JP, Schmid TL, Dower P, Hedlund S. Community heart health programs: Components, rationale, and strategies for effective interventions. Journal of Public Health Policy 1993;14(4):463-79.

(29) Ellsworth PD, Rumbaugh JH. Community organization and planning consultation: Strategies for community-wide assessment and preventative program design. Occupational Therapy in Mental Health 1980;1(1):33-55.

(30) Elwood S, Leitner H. GIS and spatial knowledge production for neighborhood revitalization: Negotiating state priorities and neighborhood visions. Journal of Urban Affairs 2003;25(2):139-57.

(31) Elwood SA. GIS and collaborative urban governance: Understanding their implications for community action and power. Urban Geography 2001;22(8):737-59.

(32) Elwood SA. GIS use in community planning: A multidimensional analysis of empowerment. Environment and Planning 2002;34(5):905-22.

(33) Fisher EB, Jr., Auslander W, Sussman L, Owens N, Jackson-Thompson J. Community organization and health promotion in minority neighborhoods. Ethnicity & Disease 1992;2(3):252-72.

(34) Fishman R. A conglomerate model for community mental health. Hospital & Community Psychiatry 1970;21(4):127.

(35) Fleury M-J. Integrated services networks: The Quebec case. Health Services Management Research 2006;19(3):153-65.

(36) Ford N, Wilson D, Bunjumnong O, von Schoen AT. The role of civil society in protecting public health over commercial interests: Lessons from Thailand. Lancet 2004;363(9408):560-3.

(37) Foster MC. Power to the people. Health Service Journal 1992;102(23 Jan 92):24-5.

(38) Fourcher LA. Compliance structures and change within mental health service organizations. Sociology of Work & Occupations 1975;2(3):246.

(39) Germann K, Wilson D. Organizational capacity for community development in regional health authorities: A conceptual model. Health Promot Internation 2004;19(2):289-98.

(40) Gómez-Jauregui J. Participation in reproductive health policies in the context of health system reform in Mexico. IDS Bulletin 2008;38(6):81-7.

(41) Green L, Daniel M, Novick L. Partnerships and coalitions for community-based research. Public Health Rep 2001;116:20-31.

(42) Greyling B, Toit A-M, Benon MC, Kuzwe CN, Reeler AP, Robertson G et al. Conflict management and peace-building through community development. Journal of Social Development in Africa 1998;13(1):7-83.

(43) Guy BN. Mental health organizations in transition. Australian and New Zealand Journal of Psychiatry 1975;9(1):9.

(44) Hardina D. Guidelines for ethical practice in community organization. Social Work 2004;49(4):595-604.

(45) Harper GW, Contreras R, Bangi A, Pedraza A. Collaborative process evaluation: Enhancing community relevance and cultural appropriateness in HIV prevention. Journal of Prevention and Intervention in the Community 2003;26(2):53-69.

(46) Hartman C. Dilemmas of community organizing: Mission Hill in Boston--the context. Social Policy 1978;9(1):41-2.

(47) Hastings E, Gorth M, Ghuman HS, Ghuman HS, Sarles RM. Child and adolescent services in a community mental health center: Transition, organization, and staffing issues. In: Ghuman HS, Sarles RM, editors. Handbook of child and adolescent outpatient, day treatment and community psychiatry.Philadelphia, PA, US: Brunner/Mazel; 1998. p. 21-31.

(48) Hein W, Kohlmorgen L. Global health governance: Conflicts on global social rights. Global Social Policy 2008;8(1):80-108.

(49) Hemman EA, McClendon BJ, Lightfoot SF. Networking for educational resources in a rural community. Journal of Continuing Education in Nursing 1995;26(4):170-3.

(50) Hennessey J, West MA. Intergroup behavior in organizations: A field test of social identity theory. Small Group Research 1999;30(3):361-82.

(51) Hood VL, Kelly B, Martinez C, Shuman S, Secker-Walker R. A Native American community initiative to prevent diabetes. Ethnicity & Health 1997;2(4):277-85.

(52) Hou J, Kinoshita I. Bridging community differences through informal processes - Reexamining participatory planning in Seattle and Matsudo. Journal of Planning Education and Research 2007;26(3):301-14.

(53) Irigoyen M, Findley SE. Methodological difficulties in assessing contributions by community-based organizations to improving child health. Archives of Pediatrics & Adolescent Medicine 1998;152(4):318-20.

(54) Kegler MC, Glanz K. Perspectives on group, organization, and community interventions. In: Glanz K, Rimer BK, Viswanath K, editors. Health Behavior and Gealth Education: Theory, Research, and Practice.San Francisco, CA, US: Jossey-Bass; 2008. p. 389-403.

(55) Lee C. Collaborative models to achieve environmental justice and healthy communities. Power, Justice, and the Environment: A Critical Appraisal of the Environmental Justice Movement.Cambridge, USA: MIT Press; 2005.

(56) Lega F. Organisational design for health integrated delivery systems: Theory and practice. Health Policy 2007;81(2-3):258-79.

(57) Livet M. Organizational characteristics, use of comprehensive programming frameworks, and programming quality: An exploratory study. Dissertation Abstracts International: Section B: The Sciences and Engineering 2007;67(9-B):5455.

(58) Longest BBJr. The community development potential of large health services organizations. Community Dev J 2006;41(1):89-103.

(59) Maccoby M. Health care organizations as collaborative learning communities. In: Adler PS, Heckscher CC, editors. The Firm as a Collaborative Community: Reconstructing Trust in the Knowledge Economy.Oxford, New York: Oxford University Press; 2006. p. 259-80.

(60) Morrison I, Schwartz P, Yankelovich D. Healthcare in the new millennium: The long boom meets the civil society. Healthcare Forum Journal 1998;41(3):18-22, 70, 72-7.

(61) Myers J. Reconceptualizing social responses to medical problems: Breast cancer and the power of community-based organizations. Dissertation Abstracts International, A: The Humanities and Social Sciences 1997;58(3):1117.

(62) Nicholls V. Contracting and the voluntary sector: A critique of the impact of markets on Mind organizations. Critical Social Policy 1997;(51):101-14.

(63) Özcan YA. Determinants of performance in community mental health organizations: A macro perspective. Dissertation Abstracts International: Section B: The Sciences and Engineering 1989;49(8-B):3098.

(64) Rand NE. Organization development: A new modality for community mental health. Am J Community Psychol 1978;6(2):157.

(65) Ratliff W. Development and civil society in Latin America and Asia. Annals of the American Academy of Political & Social Science 1999;565:91-112.

(66) Rochefort DA, Rosenberg M, White D. Community as a policy instrument: A comparative analysis. Policy Studies Journal 1998;26(3):548-68.

(67) Ruef M. The Emergence of Organizational Forms: A Community Ecology Approach. American Journal of Sociology 2000;106(3):658-714.

(68) Sladen-Dew N, Bigelow DA, Buckley R, Bornemann S. The Greater Vancouver Mental Health Service Society: 20 years' experience in urban community mental health. The Canadian Journal of Psychiatry 1993;38(5):308.

(69) Sparacino PS. An AIDS community-based organization: An innovative CNS practice model. Clinical Nurse Specialist 2000;14(1):7.

(70) Stevenson GS. Community organization for a general mental health program. American Journal of Mental Deficiency 1951;55(4):479-84.

(71) Van de Ven AH, Walker G. The dynamics of interorganizational coordination. Administrative Science Quarterly 1984;29:598-636.

(72) van Raak A, Paulus A. A sociological systems theory of interorganizational network development in health and social care. Systems Research and Behavioral Science 2001;18(3):207-24.

(73) Vogel A. Who's making global civil society: Philanthropy and US empire in world society. British Journal of Sociology 2006;57(4):635-55.

(74) Weisbrod BA. Guest Editor's Introduction: The nonprofit mission and its financing. Journal of Policy Analysis and Management 1998;17(2):165-312.

(75) Wu B, Carter MW, Goins RT, Cheng C. Emerging services for community-based long-term care in urban China: A systematic analysis of shanghai's community-based agencies. Journal of Aging & Social Policy 2005;17(4):37-60.

**Appendix 8 – References not coded**

(1) Abramovitz AJ. Interlakes Community Health Centers: A case study. Nonprofit Management and Leadership 1993;4(2):229-32.

(2) Annandale E, Tantam D, Birchwood MJ. Health services and institutions. In: Tantam D, Birchwood MJ, editors. Seminars in Psychology and the Social Sciences.London, England: Gaskell/Royal College of Psychiatrists; 1994. p. 333-51.

(3) Baum F, Fry D, Lennie I. Community health policy and practice in Australia. Sydney, Australia: Pluto Press; 1992.

(4) Chng CL, Sy FS, Choi ST, Bau I, Astudillo R. Asian and Pacific Islander American HIV community-based organizations: A nationwide survey. AIDS Education & Prevention 1998;10(3 Suppl):48-60.

(5) Czander WM. The relationship between organizational climate and bureaucracy of community health service delivery organizations. Dissertation Abstracts International: Section B: The Sciences and Engineering 1977;38(4-B):1948.

(6) da Silva SW. Organized civil society and mental health issues in Rio de Janeiro: a democratization of social space. Cadernos de Saude Publica 2001;17(4):933-9.

(7) Dougherty SJ. The generalist role in clubhouse organizations. Psychosocial Rehabilitation Journal 1994;18(1):95-108.

(8) Drabek AG. NGOs: Do we expect too much? Progress Reports on Health & Development in Southern Africa 1992;40-4.

(9) Ellsworth SL. Predictors of organizational innovation in community mental health centers. Dissertation Abstracts International: Section B: The Sciences and Engineering 1987;48(2-B):588.

(10) Englund A. Strategies for prevention: Role of voluntary and community organizations in implementation. Cancer Detection & Prevention 1986;9(5-6):413-5.

(11) Ferguson BW. From protest to programs: Neighborhood associations in a Brazilian municipality. Grassroots Development 1992;16(1):12-21.

(12) Ferrie J. Collaborative effect of networking: Theory, practice and dynamics. Dissertation Abstracts International: Section B: The Sciences and Engineering 1976;36(9-B):4663.

(13) Freudenberg N, Lee J, Silver D. How black and Latino community organizations respond to the AIDS epidemic: A case study in one New York City neighborhood. AIDS Education & Prevention 1989;1(1):12-21.

(14) Hevia RP. Community participation patterns in health programs. Boletin Medico del Hospital Infantil de Mexico 1977;34(1):239-55.

(15) Jette C. The supportive program for the community organization of the Ministry for Health and Social Services: A structural institutional form of the Quebecian Model of Social Development. Dissertation Abstracts International, A: The Humanities and Social Sciences 2006;66(7):2742.

(16) Kiley EE, Hovorka AJ. Civil society organisations and the national HIV/AIDS response in Botswana. African Journal of AIDS Research 2006;5(2):167-78.

(17) Landefeld JD. Human service organizations: A theoretical model. Dissertation Abstracts International: Section B: The Sciences and Engineering 1978;38(8-B):3941.

(18) Larose G, Vaillancourt Y, Fréchette L, Théolis M, Thomas D, Lesemann F et al. Assessing voluntary work. Nouvelles Practiques Sociales 2002;15(2):17-119.

(19) Lester EA. Alternative routes to mental health through innovations in organizational change, education and accreditation. Dissertation Abstracts International: Section B: The Sciences and Engineering 1976;36(9-B):4757.

(20) Morris RJ, Capuzzo P, Piretti S. The origins of civil society: Citizen associations in Great Britain. Contemporanea 1999;2(2):183-204.

(21) Mosley CW. Citizens Ensuring Access to Health Care (CEATH). A case study in advocacy. Nursing Leadership Forum 2002;7(1):12-5.

(22) Nair Y, Campbell C. Building partnerships to support community-led HIV/AIDS management: A case study from rural South Africa. African Journal of AIDS Research 2008;7(1):45-53.

(23) Novick RG. Community organization for mental health. Mental Hygiene 1950;34(2):203-18.

(24) Perkins JE. Community organization. American Journal of Public Health & the Nation's Health 1957;47(11 pt 2):22-4.

(25) Porter EB. Community organization; the dynamics of community action. Journal of Rehabilitation 1953;19(4):4-18.

(26) Young CL. Community organizations: A resource for rural volunteer programs. Volunteer Leader 1985;26(1):10-3.

**Appendix 9**

We documented each additional term beyond those we had included in our coding framework and grouped them thematically (see Table 1)

**Table 1: Outline of terms extracted from included studies that describe community-based organizations**

| Community coalition | Community coalition(s)/networks/partnerships (n=20) |
| --- | --- |
| Community coalition |  |
| Community coalition |  |
| Community coalition |  |
| Community coalitions |  |
| Community coalitions |  |
| Community coalitions |  |
| Community coalitions |  |
| Community coalitions |  |
| Community coalitions |  |
| Community coalitions |  |
| Community coalitions |  |
| Rural health coalitions |  |
| Inter-organizational networks |  |
| Consumer networks |  |
| Community-based coalitions |  |
| Community-based networks |  |
| Community health partnerships |  |
| Community health promotion coalitions |  |
| Coalitions |  |
| Community service networks |  |
| Community agencies | Community agencies (n=5) |
| Community agencies |  |
| Community agencies |  |
| Community agencies |  |
| Community agency |  |
| Community health and welfare agencies | Community health agencies/organizations/centres (n=17)  -Add ‘Community center’  - Add ‘Local community service center’ |
| Community health care organization |  |
| Community health center |  |
| Community health center |  |
| Community health centers |  |
| Community health centers |  |
| Community-based behavioral health providers |  |
| Community health organization |  |
| Community health promotion coalitions |  |
| Community organization |  |
| Community organization |  |
| Community organizations |  |
| Community organizations |  |
| Community planning organizations |  |
| Community centre |  |
| Local community service centers |  |
| Local community service centers |  |
| Advocacy organization | Advocacy organization (n=1) |
| AIDS service organizations | AIDS service organizations (n=1) |
| Community care access centers | Community care access centers (n=1) |
| Community development corporation | Community development corporation/organization (n=2) |
| Community development organization |  |
| Neighborhood committees | Neighborhood associations/congregations/health centers/organizations (n=9) |
| Neighborhood association |  |
| Neighborhood associations |  |
| Neighborhood congregations |  |
| Neighborhood health centers |  |
| Neighborhood health centers |  |
| Neighborhood organization |  |
| Neighborhood organizations |  |
| Cooperative neighborhood organizations* |  |
| Community governed non-profit primary care organizations | Non-profit organization/agencies/consortium/sector (n=10) |
| Non-profit agencies |  |
| Non-profit organization |  |
| Non-profit organizations |  |
| Non-profit organization |  |
| Non-profit organizations |  |
| Non-profit sector |  |
| Non-profit sector |  |
| Non-profit service consortium |  |
| Not-for-profit organizations |  |
| Cooperatives | Cooperatives (n=2) |
| Cooperative neighborhood organizations |  |
| Health service organization | Health/social service organization (n=4) |
| Human service agencies |  |
| Service organizations |  |
| Social service agencies |  |
| Consumer/survivor initiatives | Consumer/survivor initiatives (n=1) |
| Community boards | Community boards (n=1) |
| Third sector organizations | Third sector organizations (n=1) |
| Mental health organization | Mental health organization (n=1) |
| Rape crisis center | Rape crisis center (n=1) |

*Included in two categories: 1) Neighborhood associations/congregations/health centers/organizations; and 2) Cooperatives
